# The burden of chronic spontaneous urticaria in Europe: Call for action

**DOI:** 10.1111/jdv.20893

**Published:** 2025-09-26

**Authors:** Emek Kocatürk, Simon Francis Thomsen

**Affiliations:** ^1^ Institute of Allergology Charité – Universitätsmedizin Berlin, Corporate Member of Freie Universität Berlin and Humboldt‐Universität Zu Berlin Berlin Germany; ^2^ Fraunhofer Institute for Translational Medicine and Pharmacology ITMP, Immunology and Allergology Berlin Germany; ^3^ Department of Dermatology Bahçeşehir University School of Medicine Istanbul Turkey; ^4^ Department of Biomedical Sciences, Bispebjerg Hospital, Department of Dermatology University of Copenhagen Copenhagen Denmark

Chronic spontaneous urticaria (CSU) is a highly burdensome inflammatory condition that manifests with sudden onset of itch, wheals and angioedema, which causes patients to feel a loss of control and suffer from emotional distress, including anxiety, embarrassment and self‐consciousness.^1^ These symptoms significantly impair sleep, sexual function, social life and performance in school or work, and are commonly accompanied by psychiatric comorbidities such as depression and anxiety.^2^ Despite the high burden and the need for comprehensive care including effective treatment and regular follow‐up, many patients remain undertreated—for instance, a study from Germany found that 60% of CU patients were not receiving any treatment.^3^

In their recent paper, Balp et al.^4^ delve into the investigation of the prevalence, treatment approaches and overall impact of CSU in five European countries, including France, Germany, the UK, Italy and Spain. The prevalence of diagnosed CSU was 0.92%, varying by country—from the lowest in France to the highest in Italy—with a mean age of 42.4 years and 58% female predominance. The primary diagnosis was most frequently made by a primary care physician (35%), a dermatologist (35%) and an allergologist (27%). Interestingly, more than half of the patients did not report receiving any treatment for their CSU, and more than 70% reported poorly controlled urticaria even when they received treatment. The frequency of biologic treatment was <1%. This study also highlighted the significant impairment in health‐related quality of life (HRQoL) among patients, as evidenced by markedly reduced scores on different HRQoL scales, with particularly severe impacts observed in the UK. Patients showed significantly higher rates of anxiety, depression and work productivity impairment compared to the general population, with consistent findings across all countries studied. The burden was most pronounced in the UK, where CSU patients reported the highest levels of anxiety, depression, absenteeism, presenteeism and overall activity impairment. Furthermore, CSU patients utilized significantly more healthcare resources than the general population—including higher rates of emergency room visits, hospitalizations and more frequent consultations with healthcare providers—particularly specialists such as dermatologists and allergists.

The study reinforces the profound psychosocial toll of CSU, including anxiety, depression and work impairment, underscoring the need for integrated care models that address both physical and mental health. The particularly severe burden observed in the UK raises questions about country‐specific healthcare system differences, patient access to care or socio‐cultural factors influencing disease perception and management outcomes.

These findings highlight a major gap between high disease burden and under‐treatment across Europe and very low utilization of biological therapies despite established guidelines^5^ and well‐established reimbursement strategies for omalizumab which suggest significant barriers to access prescribing practices. On the other hand, the large proportion of diagnoses made by primary care physicians raises concerns about awareness, training and confidence in CSU management at the primary care level. To address this, targeted educational initiatives for general practitioners, clearer referral guidelines and the integration of structured pathways are needed that will ensure timely access to specialist care and proper management.

The current state of CSU management in Europe reveals a striking disconnect between disease burden and quality of care. This situation demands immediate policy‐level attention and system‐wide changes. National health systems must take CSU seriously—not only as a simple condition but also as a complex, chronic disease with substantial physical, psychological and socio‐economic consequences. Concrete measures are needed, including raising awareness among healthcare providers and policymakers, improving access to guideline‐recommended treatments (particularly biologics), investing in primary care training and implementing structured referral and management pathways (Figure 1). Only through coordinated and sustained action can we ensure that CSU patients receive the care they need and deserve.

**FIGURE 1:**
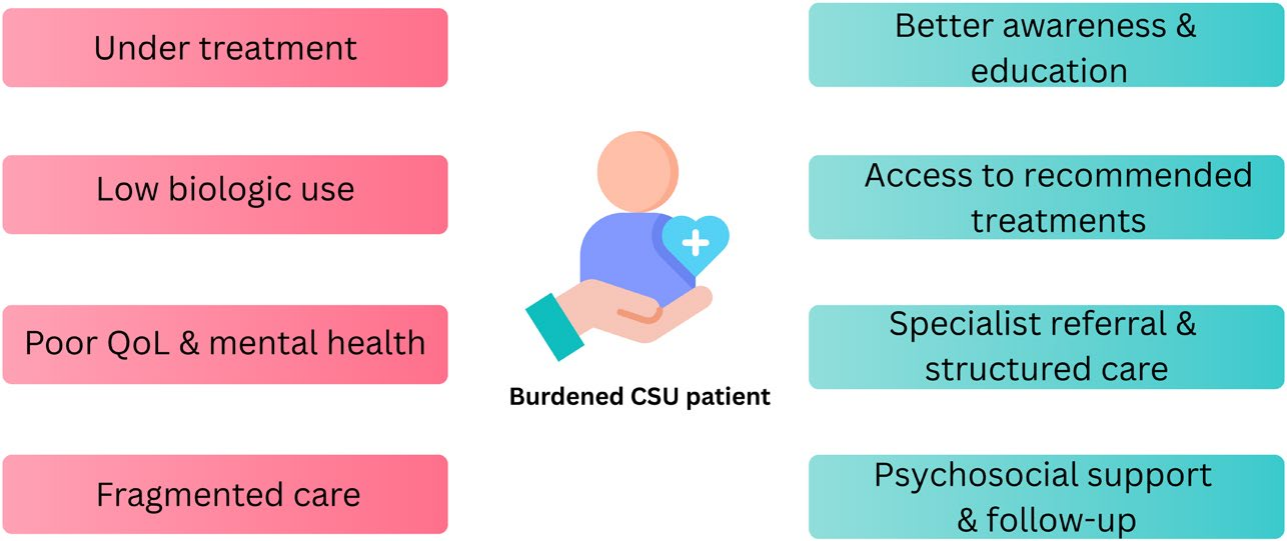
Bridging the gap in CSU care: from burden to solution.

## CONFLICT OF INTEREST STATEMENT

EK has acted as a speaker or advisor for Novartis and Menarini and received funding from Almirall. SFT is or recently was a speaker or advisor for AbbVie, Almirall, Boehringer, Eli Lilly, Galderma, Incyte, Janssen Pharmaceuticals, LEO Pharma, Novartis, Pfizer, Sanofi, UCB Pharma and Union Therapeutics and received research support from AbbVie, Janssen Pharmaceuticals, LEO Pharma, Novartis, Sanofi and UCB Pharma, outside the submitted work.

## Data Availability

Data sharing is not applicable to this article as no new data were created or analysed in this study.
